# Neonatal thrush of newborns: Oral candidiasis?

**DOI:** 10.1002/cre2.213

**Published:** 2019-07-21

**Authors:** Anne Vainionpää, Jenni Tuomi, Saara Kantola, Vuokko Anttonen

**Affiliations:** ^1^ Research Unit of Oral Health Sciences University of Oulu Oulu Finland; ^2^ Department of Special Dentistry Unit Oral Health Care of City of Oulu Oulu Finland; ^3^ Department of Oral and Maxillofacial Diseases Oulu University Hospital Oulu Finland; ^4^ Medical Research Center Oulu University Hospital and University of Oulu Oulu Finland

**Keywords:** Candida albicans, infant, newborn, oral candidiasis, thrush

## Abstract

**Objectives:**

Neonatal thrush, also called oral candidiasis, is commonly a clinical diagnosis based on white patches on oral mucosal surfaces. Candida albicans is often associated with it. This clinical study aimed to investigate the presence of C. albicans among newborns with or without clinical findings of candidiasis on oral mucosa. Another aim was to investigate how thrush responded to current therapy by acidic liquids such as lingonberry or lemon juice.

**Material and methods:**

Swipe samples were collected from 32 healthy, full‐term infants younger than 12 months with or without white patches on oral mucosa. Clinical diagnosis of thrush was made by a community nurse based on thick and yellowish white patches. The routine therapy was oral lingonberry or lemon juice or soda water. Disappearing of patches was controlled by a phone call about 2 weeks after the baseline. Both parents and nurses gave background factors by filling a questionnaire.

**Results:**

One (3%) infant without clinical signs was diagnosed with Candida parapsilosis, none with C. albicans. Thrush resembling candidiasis was diagnosed clinically in four (12.5%) children. Three out of four parents reported persisting findings after 2 weeks. Only the maternal mastitis and use of antibiotics were significantly associated with thrush (*p* = .001). C. albicans was not discovered from babies with clinical thrush.

**Conclusions:**

Aetiology of the white patches remained unclear. The current way of treating them with acidic liquids is not efficient. Additional studies are needed.

## 1 INTRODUCTION

White patches on an infant's (generally under 1 year) tongue and/or oral mucosa are commonly called neonatal thrush and referred to as oral candidiasis (Baley, 1991; Dobias, 1957). The prevalence varies between 4% and 15% in literature (Dobias, 1957; Issa, Badran, Aqel, & Shehabi, 2011; Kozinn, Taschdjian, Wiener, Dragutsky, & Minsky, 1958; Stecksen‐Blicks, Granström, Silvferdal, & West, 2015). Candida albicans (*CA*) is the most common oral yeast and associates with thrush. *CA* colonises the newborn either vertically during vaginal birth or horizontally from the environment (Filippidi et al., 2014; Stecksen‐Blicks et al., 2015). Thrush usually disappears by itself, but medications are prescribed for difficult symptoms (Harris, Pritzker, Eisen, Steiner, & Shack, 1958). In Finland, oral drops of lingonberry/lemon juice are used for treating thrush. All newborns are checked in community clinics monthly until 6 months and after that every 2 months until the child turns 1 year. This offers an ideal possibility for monitoring thrush.

Literature on the aetiology of thrush among healthy, full‐term infants is scarce. This study aimed to investigate the presence of *CA* among healthy Finnish infants younger than 1 year with or without clinical signs of thrush. Another aim was to investigate the response to local therapy by lingonberry/lemon juice or soda water.

## 2 MATERIAL AND METHODS

Swipe samples were collected from 32 full‐term, healthy infants younger than 1 year with or without thrush. They came for a routine check‐up visit in a community clinic in Ii, Finland. Mean age of the participants was 4.8 months (*SD* 3.13). The community nurses (*n* = 3) acquainted with taking swipe samples, took the sample of oral mucosa by a cotton stick (Transystem M40, plastic applicator w/o Charcoal). Samples were cultured for Candida fungi according to the protocol of the Northern Ostrobothnia Hospital District, Finland.

The nurses recorded clinically white patches as thrush, if they appeared thick and yellowish excluding thinner white milk patches. Parents were instructed to drop lingonberry or lemon juice or soda water on infant's oral mucosa if thrush was present. Disappearing of thrush was controlled by a phone call about 2 weeks after the baseline.

At baseline nurses recorded the age of a child (months), sample site, oral findings (thrush/no thrush), and treatment, if needed. According to contacting parents by phone in 2 weeks, they recorded persisting white patches (y/n). The parents gave background factors about gestation time (months) and way of delivery (natural/Caesarean section), the main way of feeding (bottle/breast), contents of bottle (maternal milk/infant formula/water/juice/something else), use of pacifier (y/n), eruption time of the first deciduous tooth (months), mother's dental treatment (open question), child's possible otitis media (AOM, y/n) and use of antibiotics, mother's mastitis, and her use of antibiotics (y/n).

The community clinic agreed to participate in collecting data. A written consent was obtained from all parents and the study was conducted according to the Declaration of Helsinki of the World's Medical Association. The study protocol has been reviewed and approved by the ethical board of Northern Ostrobothnia Hospital District (43 §/2017).

Presence of thrush in the study population was presented as proportions. Association between the clinical signs and cultured Candida as well as background factors was calculated by cross tabulation and analysed chi‐squared test; *p* values < .05 were considered statistically significant. All analyses were executed with the SPSS (version 24.0, SPSS, Inc., Chicago, IL, USA).

## 3 RESULTS

Thrush was clinically detected in four children (12.5%), yet no *CA* was detected in them, or any other samples. One (3%) infant was diagnosed having Candida parapsilosis (*CP*). Maternal mastitis and use of antibiotics were significantly associated with clinical thrush findings (*p* = .001). In three cases with white patches on oral mucosa at the baseline, the condition remained after 2 weeks despite the treatment.

## 4 DISCUSSION

Clinically diagnosed thrush among healthy full‐term infants does not correspond with culturable findings (12.5%/none, respectively). *CA* was not detected in any sample here, which is contradictory to earlier findings (Issa et al., 2011; Stecksen‐Blicks et al., 2015). *CP* was found in one sample, yet without clinical findings. *CP* comprises one third of all Candida infections specifically among preterm neonates (Pammi, Holland, Butler, Gacser, & Bliss, 2013).

Only maternal mastitis and use of antibiotics were associated with thrush here. In literature *CA* colonisation has been associated with siblings as a predisposing and family pets as a protective factor (Stecksen‐Blicks et al., 2015).

Despite the treatment, white patches persisted in three out of four cases. Lingonberry and lemon juice basing on their acidity (pH 2.7 and 3.1, respectively), traditionally used in Finland to treat thrush, don't seem effective. Maternal mastitis and use of antibiotics may indicate the aetiology of some other microbes. Thrush was not *CA* infection, so, the question remains, what is it: milk, detaching mucous membrane or perhaps some other microbes. Additional studies to identify the composing elements of white patches are needed. There were no clinical pictures, which would have been valuable, and the study population was small, therefore these results must be considered preliminary.

## CONFLICT OF INTEREST

A.V. is supported with a grant by The Finnish Dental Society Apollonia.
